# Barriers to COVID-19 Intervention Implementation in K-5 Classrooms: A Survey of Teachers from a District with Mask Mandates despite a Statewide Mask Mandate Ban

**DOI:** 10.3390/ijerph19148311

**Published:** 2022-07-07

**Authors:** Amanda M. Wilson, Olusola O. Ogunseye, Olivia DiGioia, Lynn B. Gerald, Ashley A. Lowe

**Affiliations:** 1 Department of Community, Environment and Policy, Mel and Enid Zuckerman College of Public Health, University of Arizona, Tucson, AZ 85721, USA; olusolaogunseye@email.arizona.edu; 2 Department of Health Promotion Sciences, Mel and Enid Zuckerman College of Public Health, University of Arizona, Tucson, AZ 85721, USA; odigioia@email.arizona.edu (O.D.); lgerald@arizona.edu (L.B.G.); 3 Asthma & Airway Disease Research Center, Tucson, AZ 85724, USA; aaray@arizona.edu

**Keywords:** school health, pandemic, emergency preparedness

## Abstract

The study objective was to characterize K-5 teachers’ risk perceptions and experiences with CDC COVID-19 classroom guidance in an Arizona school district with a mask mandate, conflicting with a statewide mask mandate ban. Methods: Public school teachers (*n* = 111) were recruited between 14 December 2021, and 31 January 2022, for an anonymous online survey with questions on seven important topics related to: (1) population demographics, (2) teachers’ perceptions of COVID-19 in the workplace, (3) masks, (4) physical distancing, (5) surface transmission routes, (6) air flow, and (7) contact tracing protocols. Descriptive statistics were calculated, and statistically significant differences in categorical responses by grade level taught were investigated with Fisher’s exact test. Results: There were 76 complete responses. No significant differences across grade levels were found. More than half (53%, 43/81) reported not feeling protected from occupational COVID-19 exposure. Lack of mask usage/enforcement was the most frequently listed reason (40%, 17/42). Physical distancing barriers included large student-teacher ratios. Conclusions: Consistent mask guidance at state and local levels, increased financial support, and lower student-teacher ratios may improve the implementation of CDC guidance for classrooms. Conflicting statewide and district-level school mask policies may negatively impact teachers’ risk perceptions.

## 1. Introduction

The COVID-19 pandemic has highlighted the burdens of stress and health risks placed on frontline workers. One of the most impacted occupations has been elementary school teachers, who are responsible for keeping both staff and students safe. In an investigation of COVID-19 school outbreaks in the United Kingdom, 73% of outbreaks originated from an infected staff member [1]. To protect students and staff in schools, the Centers for Disease Control and Prevention (CDC) issued school-specific guidance for K-12 schools as the pandemic progressed. The CDC issued a warning to schools on 25 February 2020 to prepare for school closures and virtual learning plans and provided guidance in Summer 2020 to prepare for students returning for Fall 2020 [2,3]. This guidance was updated as more age groups became eligible for vaccinations, and it includes information on vaccine promotion, mask use, ventilation, physical distancing, testing, contact tracing, and surface and hand hygiene [4].

Lack of compliance with mitigation guidelines can lead to school outbreaks [5]. The odds of COVID-19 outbreaks occurring in schools without mask requirements were 3.5 times higher (95% CI: 1.8–6.9) than those with mask requirements [6]. In a school outbreak in Israel, the absence of mask enforcement, lack of physical distancing, and use of recirculating air conditioning in response to a heat wave were contributing factors, leading to an attack rate of 16.6% among educational staff [5]. Given individual perceptions surrounding key interventions (i.e., masks), and the lack of resources for implementing more costly interventions (i.e., environmental mitigation such as improved ventilation systems), the layering [7] of effective strategies remains necessary in schools [8]. During Israel’s outbreak, physical distancing was not maintained between students because it “was not possible” [5].

The stress and organizational challenges and feelings of burnout among teachers internationally are widely recognized [9,10,11,12,13]. In the U.S., specifically, the real-world challenges for teachers in implementing CDC guidance for K-12 schools are not well characterized. Inventorying barriers to the implementation of these approaches will inform current and future pandemic guidance and outbreak preparedness, which will ideally consider real-world scenarios (i.e., lack of resources, political conflict, logistic obstacles, and risk perceptions). The objective of this study was to collect data on teachers’ risk perceptions and experiences with the implementation of CDC guidance during the COVID-19 pandemic in the 2020–2021 and 2021–2022 school years. The pilot data described here are from K-5 teachers in Tucson, Arizona.

## 2. Materials and Methods

Participants were recruited between 14 December 2021 and 31 January 2022 from a public school district in Tucson, Arizona, which contains 22 schools, approximately 552 K-5 teachers, and 14,942 students. This school district implemented a mask mandate from 19 August 2021 to 21 March 2022 (references not included to protect district identity), in direct conflict with Arizona law that outlawed mask mandates. State funds were withheld from schools that implemented mask mandates [14].

All teachers of grades K-5 who were teaching in person were included. We focused on K-5 teachers since large differences in guidance implementation barriers were expected between K-5, middle school, and high schools, and vaccines were not rolled out for children of ages expected for K-5 grades (5–11 years old) until 8 November 2021. Information about the study, a link to consent, and an online anonymous survey were distributed to teachers via email through school district personnel. Schools with participating teachers were compensated for their participation with a free teacher lunch. This study was approved and monitored by the University of Arizona Institutional Review Board (protocol #: 2107972830) and by the participating school district administration.

### 2.1. Survey

An anonymous online survey built in Research Electronic Data Capture (REDCap) [15], used for data collection and management and hosted at the University of Arizona, was developed through discussion among school health, occupational, and environmental health experts and in partnership with the school district in which the survey would be disseminated. The school district reviewed, revised, and approved the final survey instrument. The survey contained questions on seven important topics related to: (1) population demographics, (2) teachers’ perceptions of COVID-19 in the workplace, (3) masks, (4) physical distancing, (5) surface transmission routes, (6) air flow, and (7) contact tracing protocols. The survey took approximately 30 min to complete and was administered through REDCap. The survey tool is available in Supplementary Materials.

### 2.2. Survey Analysis

Descriptive statistics were calculated for participant demographics, including age, gender, and information on the schools at which they teach, including the number of students attending their school (2021–2022 school year), grades taught in the 2021–2022 school year, and whether the school has a designated health office. Fisher’s exact tests (α = 0.05) were used to evaluate statistically significant differences in categorical responses across grade levels taught. When there were statistically significant differences, descriptive statistics were stratified. All statistical analysis was completed using R statistical software [16] (version 4.1.0, R Core Team, R Foundation for Statistical Computing, Vienna, Austria) and STATA (version 16, StataCorp LLC, College Station, TX, USA) [17].

Short-answer responses were analyzed by a researcher for the frequency of specific responses listed (e.g., types of barriers listed for physical distancing in the classroom) and for quotes that represented differences in the perception of COVID-19 risks or barriers. Our team used a deductive approach [18], which relies on general statements and participant responses to generate themes. The themes used for analyzing reasons for feeling unprotected from COVID-19 exposure at work included lack of masks, inconsistent protocols when someone tests positive, lack of social distancing, lack of cleaning, lack of hand hygiene, and lack of ventilation. The themes used for analyzing physical distancing barriers in classrooms included class size/not enough space, furniture type/use of workstations, and type of class (i.e., instructional challenges). For overall comments provided by teachers, two researchers selected quotes to expand upon the breadth and range of the survey responses and to highlight the most representative opinions reflected in the survey responses. 

## 3. Results

### 3.1. Participant Demographics

There were 111 responses, but only 76 were complete. Nine additional surveys had nearly complete responses, yielding nearly 85 completed responses. This translates to a 15% (85/552) response rate. A low response rate was anticipated, given the strain on teachers during the COVID-19 pandemic. The median age of teachers and median years of experience were 44 (IQR: 19) and 11 years (IQR: 14), respectively (Table 1). A majority of the teachers (81%, 69/85) were female. Thirty-nine percent of teachers (33/85) reported the number of students that attended their school at the beginning of the 2021–2022 school year ranged from 501–750. Thirty-seven percent of teachers (36/97) reported that they teach special topics (e.g., physical education, music) for all ages (K-5). All teachers (100%, 85/85) reported that their schools have a designated health office or isolation space for sick children. Over half of the teachers (59%, 45/76) indicated that the total number of students they teach in the classroom per day in the 2021–2022 school year was between 21–40 students. About half of the teachers (51%, 39/76) reported that the total number of students they teach in the classroom per week was more than 40 students. No statistically significant differences in response based on the grade level taught were observed. Responses were therefore combined across grade levels taught. 

### 3.2. Teachers’ Perceptions of COVID-19 in the Work Place

Over half of the teachers (53%, 43/81) reported not feeling protected from exposure to COVID-19 in the workplace, even though 90% (73/81) had received at least one dose of the COVID-19 vaccine. Thirty-five percent (28/81) reported having received a positive diagnostic test for COVID-19 since March 2020. For those who did report feeling protected at work, the most stated reason (40%, 17/42) was mask usage or enforcement, followed by lack of social distancing (33%, 14/42) and not being notified when a student tests positive or students/staff coming to school sick (31%, 13/42). There were mixed overall sentiments shared by teachers. One teacher stated, “I’ve been fortunate that all of my students and families are cooperative about safety measures,” while another stated, “These have been the worst years I’ve ever taught”.

### 3.3. Masks

**Figure 1:**
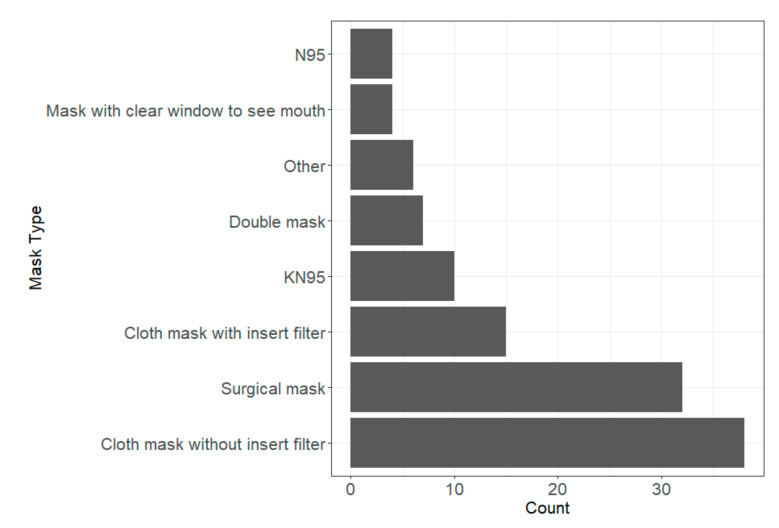
Count of mask types used by teacher participants during the 2020–2021 school year. Total count can be greater than the total number of participants, as participants could select more than one mask type.

The most used mask type was a cloth mask without an inserted filter, followed by surgical masks (Figure 1). Of those who reported using N95s (four participants), none had been fit tested. When asked to think back on the 2020–2021 school year, 81% (64/79) of teachers reported their masks fitting well. A majority of teachers did not feel protected when they were not wearing masks (60%, 47/78) and when students were not wearing masks (64%, 49/77). Fifty-four percent (43/79) reported having issues with mask compliance in the classroom. While 72% (56/78) reported some masks being provided by their school, 60% (47/78) reported having to use personal funds to purchase extra masks for the classroom.

### 3.4. Physical Distancing & Shared Staff Spaces

Fifty-eight percent of teachers (44/76) reported not having enough space in their classroom to maintain 3 ft physical distance between students. For those who included reasons (*n* = 43), barriers to maintaining 3 feet of distance included large class sizes and physical constraints (79%, 34/43), furniture type (e.g., use of tables as opposed to desks) (35%, 15/43), and teaching-specific limitations (5%, 2/43) (e.g., close contact required for dance or some students needing to sit closer to the board).

### 3.5. Surface Transmission Routes

Most teachers (93%, 71/76) reported cleaning or disinfecting their classrooms, using products including disinfectants provided by the school; wipes and sprays; anti-viral products; and products with active ingredients including bleach-, hydrogen peroxide-, benzalkonium chloride-, dimethyl benzyl ammonium chlorine, and glycolic acid-based products; and soap and water. Sixty-five percent (46/71) reported cleaning surfaces 1–2 times per day, while 15% (11/71) and 11% (8/71) reported cleaning 3–4 times or >4 times a day, respectively. 

### 3.6. Air Flow

**Figure 2:**
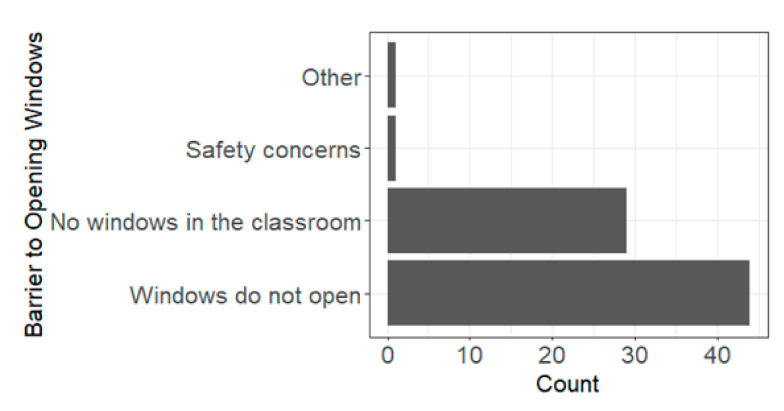
Barriers to opening windows during class time. Total count can be greater than the total number of participants, as participants could select more than one barrier to opening doors or windows.

**Figure 3:**
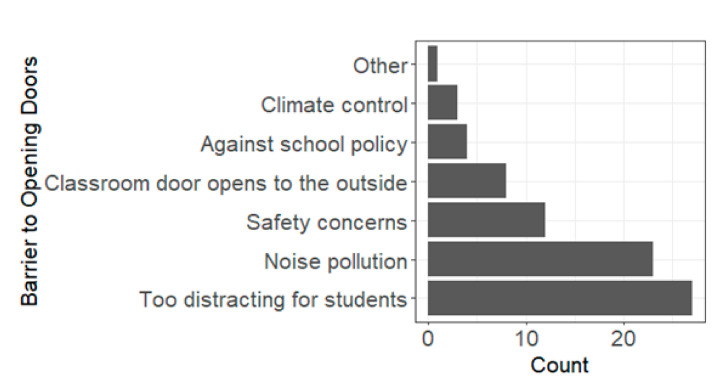
Barriers to opening doors during class time. Total count can be greater than the total number of participants, as participants could select more than one barrier to opening doors or windows.

Nearly all teachers (97%, 74/76) reported not opening windows in the classroom during the 2021–2022 school year. The most stated reason for this was having windows that do not open, followed by not having windows in the classroom at all (Figure 2). One teacher who selected “other” (Figure 2) reported issues with leaking rainwater and rotten wood around the windowpane. Regarding opening classroom doors, 57% (43/76) reported not opening doors during classes, 34% (26/76) reported sometimes, and 9% (7/76) reported yes. The most-reported reason for not opening doors was it being too distracting for students, followed by noise pollution and safety concerns (Figure 3).

### 3.7. Contact Tracing Protocols

Eighty-nine percent (67/75) of teachers reported that their schools implemented a contact tracing protocol during the 2021–2022 school year, and all teachers reported having a designated health office for sick children to go. However, 53% (40/75) reported having a separate place for children suspected of having COVID-19, while 25% (19/75) reported not having a separate space.

## 4. Discussion

### 4.1. Summary of Findings

Despite a mask mandate being in place in this school district during a time in which mask mandates were banned in the state, more than half of the teachers (53%, 43/81) reported not feeling protected from exposure to COVID-19 in the workplace. This was also despite 90% (73/81) having received at least one dose of the COVID-19 vaccine. For those who did not feel protected at work, lack of mask usage/enforcement was the most frequently listed reason (40%, 17/42). Identified barriers to implementing CDC guidance included lack of supplies (e.g., masks) and large student–teacher ratios that made physical distancing infeasible. Other physical barriers included the use of furniture that does not allow for physical distancing (e.g., tables instead of individual desks). Logistical barriers related to instruction and classroom management were also identified, including not being able to increase airflow by opening doors due to potential distractions for students, challenges in handling mask compliance in the classroom, and masks making it difficult to teach specific subjects, such as phonics. Some teachers expressed an overall positive sentiment about their job during the pandemic, “I’ve been fortunate that all of my students and families are cooperative about safety measures,” while others expressed a negative experience, “These have been the worst years I’ve ever taught.”

### 4.2. Findings in Context

This study was conducted in Tucson, Arizona (AZ), a unique location for studying these challenges due to several key events that presented AZ schools with additional challenges: Governor Doug Ducey only gave funds from the $173 million American Rescue Plan aid to schools that did not implement COVID-19 mask mandates [19]. This decision was based on the passage of a law banning mask mandates (effective on 29 September 2021) [14], which the Arizona Supreme Court later ruled was illegal [20]. Despite these events, the school district in this study maintained their mask mandate. In the U.S. as of 29 March 2022, 5 states have mask mandate bans in effect (Florida, Georgia, Oklahoma, Utah, and Virginia), and 6 states have mask mandate bans that have been blocked, suspended or unenforced (Arizona, Arkansas, Iowa, South Carolina, Tennessee, Texas) [21]. Only 1 state has a mask requirement in effect (Hawaii), and 18, including District of Washington, previously had a mask requirement in effect [21].

In addition to having an impact on teachers’ perceived risks, masks are effective at reducing aerosol emissions and exposure [22]. Pan et al. (2021) found that three of eleven tested material types yielded material filtration efficiencies that were greater than 50% (vacuum bag, microfiber cloth, single-layer surgical-type mask) for 0.3 μm, the size posing the most penetration [22]. The most reported mask type used by teachers in this study was a cloth mask without a filter (Figure 1). While this mask type does offer some protection (common fabrics providing approximately 40% efficiency for 1 μm particles), this protection could be improved through the use of N95s or three-layered masks with two layers of a “flexible, tightly woven fabric” and a most inward layer made of a filtration material, such as MERV 14 [22].

The fact that identified barriers included infrastructural problems (e.g., not being able to open windows, large student to teacher ratios and lack of space for physical distancing) was not surprising. The physical infrastructural challenges that schools face due to aging buildings is widely recognized, especially for students in low-income communities [23]. There are health disparities related to COVID-19 risks across schools [24], where districts serving low-income households likely have greater infrastructural barriers in protecting staff and students from COVID-19 exposures, in part due to limited financial resources. In this study, sixty percent (47/78) reported having to use personal funds to purchase extra masks for the classroom. Future research is needed to elucidate differences in barriers to the implementation of CDC guidance across school districts with differing budgets and student body demographics. 

This study contributes to current literature on the effects of the COVID-19 pandemic on teachers, where most studies have focused on the psychological stress among teachers [11,25] and/or the impact of remote learning on education [10,26]. Fukuda and Fukuda (2022) found that one of the contributions to educators’ stress included infection prevention [25]. In a study of Denmark teachers, access to necessary personal protective equipment (PPE) and emotional responses to the implementation (or lack thereof) of infection control measures in classrooms were investigated. Nabe-Nielsen et al. (2021) found that lack of access to PPE and having close contacts with infected individuals had an association with greater emotional reactions, similar to our finding that lack of mask enforcement influenced teachers’ feelings of lack of protection from COVID-19 exposure.

### 4.3. Limitations

While the inclusion of only one school district in this study was conducted to rapidly gather data to inform pandemic guidance, this is a limitation in that the results only reflect perspectives from one set of schools in one geographical area (Tucson, AZ, USA). Organizational challenges are likely to vary depending on district administrations and how CDC guidance was implemented. Political challenges are likely to vary among schools in other states. While differences across grade levels were not seen in this study, this is potentially due to a low number of participants in each grade level category (K-5 grades and special classes, such as music) and the limited number of grade levels included. More data are needed from schools across the state and country to confirm the generalizability of the key barriers characterized in this study (i.e., lack of funding and physical space, large student-to-teacher ratios, logistical challenges with maintaining mask mandates or increasing airflow with natural ventilation).

Other biases include recall bias [27] and social desirability bias [28]. It is possible that teachers misremembered challenges in their classrooms or that developments during the pandemic have changed their perceptions from what they were previously. For example, 89% of teachers reported a contact tracing protocol being implemented in their school, even though the true proportion of schools was 100% [6]. Teachers could have also misinterpreted some of the questions, such as those pertaining to mask choices and understanding the differences between KN95s and N95s. Teachers may have misreported mask usage or other strategies to appear in compliance with CDC guidance or in line with the political opinions of their communities. This bias was minimized by using an anonymous survey to encourage teachers to answer freely, but since it was distributed through the school administration, it still likely had an influence, meaning adherence to guidelines or negative sentiments about guidelines may be underestimated. 

Another potential bias is the healthy worker effect [29], where teachers available to participate in this study may represent those most likely to continue working through barriers encountered in the workplace or who were able to maintain their health through the pandemic. This may mean that barriers and challenges encountered by teachers captured in this study underestimate the true burden that teachers faced during this time, especially considering low teacher retention during the pandemic [30].

## 5. Conclusions

The barriers teachers have faced in implementing CDC pandemic guidance shed light on needed financial support for supplies and lower student-teacher ratios along with considerations of limited furniture or curriculum-specific needs that challenge guidance implementation. Barriers to implementing guidance may be directly related to feelings of protection (or lack thereof) in the workplace, which in this case was related to feelings of being unprotected regarding lack of mask enforcement. More data are needed to characterize U.S.-wide barriers and those that were geographically specific.

## Figures and Tables

**Table 1:** Participant demographics, teachers’ perception of COVID-19 in the workplace, and their testing/vaccine status collected in Tucson, AZ (14 December 2021–31 January 2022).

Demographic Variable		Median (IQR) or % (Count/*n*)
Age (years)		44 (19)
Number of Years of Teaching Experience		11 (14)
Gender	Male	19% (16/85)
	Female	81% (69/85)
	Nonbinary	0% (0/85)
Number of Students Attending School at Beginning of the 2021–2022 School Year	>1000	9% (8/85)
	751–1000	14% (12/85)
	501–750	39% (33/85)
	251–500	26% (22/85)
	1–250	5% (4/85)
	Declined to answer/Do not know	7% (6/85)
Grade Taught at Beginning of the 2021–2022 School Year	Kindergarten	12% (12/97)
	1st	8% (8/97)
	2nd	8% (8/97)
	3rd	10% (10/97)
	4th	8% (8/97)
	5th	15% (15/97)
	Other (Special topics for all ages, such as Music or Physical Education)	37% (36/97)
Teaching at School with Designated Health Office or Isolation Space for Sick Children	Yes	100% (85/85)
	No	0% (0/85)
Total Number of Students Taught in Classroom per Day in the 2021–2022 School Year	<15 students	7% (5/76)
	15–20 students	13% (10/76)
	21–40 students	59% (45/76)
	>40 students	21% (16/76)
Total Number of Students Taught in Classroom per Week in the 2021–2022 School Year	<15 students	4% (3/76)
	15–20 students	7% (5/76)
	21–40 students	38% (29/76)
	>40 students	51% (39/76)
Feel protected from exposure to COVID-19 at work	Yes	42% (34/81)
	No	53% (43/81)
	Declined to answer/Do not know	5% (4/81)
Received at least 1 dose of COVID-19 vaccine	Yes	90% (73/81)
	No	7% (6/81)
	Declined to answer/Do not know	3% (2/81)
Feel they understand current CDC guidelines for reducing COVID-19 transmission risk in the classroom	Yes	83% (67/81)
	No	10% (8/81)
	Declined to answer/Do not know	7% (6/81)
Know who to ask at school for questions/concerns regarding COVID-19 guidance	Yes	90% (73/81)
	No	7% (6/81)
	Declined to answer/Do not know	3% (2/81)
Immediately notified by a supervisor when a student they interacted with tests positive for COVID-19	Yes	64% (52/81)
	No	30% (24/81)
	Declined to answer/Do not know	6% (5/81)

## Data Availability

The data that support the findings of this study are available from the corresponding author, A.M.W., upon reasonable request.

## Supplementary Materials

The following supporting information can be downloaded at: https://www.mdpi.com/article/10.3390/ijerph19148311/s1, to access the Survey Instrument.
