# Supplementary material for: Barriers to COVID-19 Intervention Implementation in K-5 Classrooms: A Survey of Teachers from a District with Mask Mandates despite a Statewide Mask Mandate Ban
Source: Int J Environ Res Public Health. 2022 Jul 7;19(14):8311. doi: 10.3390/ijerph19148311 (PMC9316596; doi:10.3390/ijerph19148311)
Supplement: Supplementary file 1 [file ijerph-19-08311-s001.zip › ijerph-1783218-supplementary.pdf]

Section A: Population characteristics.

Survey Instructions: The following section will ask you questions about your demographic background including gender, current age and aspects of your teaching experience. Please answer each question to the best of your ability.

What is the gender that you identify with?<br>{[gender] radio}<br>{Branching logic (show if): [consent] = '1'}	◯ {3} Male<br>◯ {2} Female<br>◯ {1} Non-binary (X)<br>◯ {0} Declined to answer<br>(Please select only one answer.)
What is your current age? (Years)<br>{[age] text float}<br>{Branching logic (show if): [consent] = '1'}	((Please write declined to answer if you do not want to disclose your age. Use numbers only please.))
What was the approximate total number of students who attended your school at the beginning of the 2021-2022 school year?<br>{[no_students] radio}<br>{Branching logic (show if): [consent] = '1'}	◯ {1} >1,000 (Students)<br>◯ {2} 751- 1,000 (Students)<br>◯ {3} 501-750 (Students)<br>◯ {4} 251-500 (Students)<br>◯ {5} 1-250 (Students)<br>◯ {6} Do not know / Declined to answer ((Students))
At the beginning of the 2021-2022 school year, what was the grade-level you primarily taught/teach during your typical work week?<br>{[grades] checkbox}<br>{Branching logic (show if): [consent] = '1'}	☐ {6} Kindergarten<br>☐ {5} First grade<br>☐ {4} Second grade<br>☐ {3} Third grade<br>☐ {2} Fourth grade<br>☐ {1} Fifth grade<br>☐ {0} Other (Special topics for all ages. Examples include Music of Physical Education)<br>(Please select all that apply.)
If other, please describe the primary subject that you spend most of your time teaching during the 2021-2022 school year.<br>{[grades_other] text}<br>{Branching logic (show if): [consent] = '1' and [grades(0)] = '1'}	(Please describe.)
Do you typically rotate between multiple classrooms throughout a typical school day?<br>{[rotate] radio}<br>{Branching logic (show if): [consent] = '1'}	◯ {1} Yes<br>◯ {0} No<br>◯ {-1} Do not know / Declined to answer<br>(Please select only one answer.)
If yes, please describe your typical teaching environment.<br>{[rotate_other] textarea}<br>{Branching logic (show if): [rotate] = '1' and [consent] = '1'}	(Please describe.)
At the beginning of the 2021-2022 school year (this school year), how many total years of teaching experience do you have?<br>{[years] textarea}<br>{Branching logic (show if): [consent] = '1'}	((Years))

Does your school have a licensed nurse (RN, MSN or DNP) that takes care of student health needs?<br>{[nurse] radio}<br>{Branching logic (show if): [consent] = '1' and [share] = '1'}	◯ {1} Yes<br>◯ {0} No<br>◯ {-1} Do not know / Declined to answer<br>(Please select only one answer.)
Does your school have a designated health office or space where children can go when they are feeling ill?<br><br>{[healthoffice] radio}<br>{Branching logic (show if): [consent] = '1'}	◯ {1} Yes<br>◯ {0} No<br>◯ {-1} Do not know / Declined to answer<br>(Please select only one answer.)

Section B: Teacher's Perception of Covid-19 in the work place.

We are interested in learning more about your personal beliefs and perceptions regarding Covid-19 and how it affected your teaching of elementary school children during the Covid-19 pandemic where physical distancing could not always be achieved. Please answer each question to the best of your ability. All survey responses will be kept confidential and aggregated with other survey responses as to not personally identify you.

Do you feel protected from exposure to Covid-19 at work (i.e., school)?<br>{[safe] radio}<br>{Branching logic (show if): [consent] = '1'}	◯ {1} Yes<br>◯ {0} No<br>◯ {-1} Do not know / Declined to answer<br>(Please select only one answer.)
If yes, what things at your school made you feel protected?<br>{[safe yes] textarea}<br>{Branching logic (show if): [safe] = '1'}	(Please describe.)
If no, what things at your school did not make you feel protected?<br>{[safe no] textarea}<br>{Branching logic (show if): [safe] = '0'}	(Please describe.)
Have you received at least 1 dose of a Covid-19 vaccine (e.g., Moderna, Pfizer or Johnson and Johnson)<br>{[vaccine] radio}<br>{Branching logic (show if): [consent] = '1'}	◯ {1} Yes<br>◯ {0} No<br>◯ {-1} Do not know / Declined to answer<br>(Please select only one answer.)
If no, do you plan on getting the Covid-19 vaccination in the future?<br>{[vaccine no] radio}<br>{Branching logic (show if): [vaccine] = '0' and [consent] = '1'}	◯ {1} Yes<br>◯ {0} No<br>◯ {-1} Do not know / Declined to answer<br>(Please select only one answer.)
If no, what sort of things have influenced your decision to not get the Covid-19 vaccination?<br>{[vaccine no more] textarea}<br>{Branching logic (show if): [vaccine no] = '0' and [consent] = '1'}	(Please describe.)
Since March 2020, did you receive a positive diagnostic test for COVID-19?<br>{[positive] radio}<br>{Branching logic (show if): [consent] = '1'}	◯ {1} Yes<br>◯ {0} No<br>◯ {-1} Do not know / Declined to answer<br>(Please select only one answer.)
If yes, approximately what month and year did you receive a positive test?<br>{[positive time] text}<br>{Branching logic (show if): [positive] = '1' and [consent] = '1'}	(Please describe.)
If yes, where do you think you may have been exposed to Sars-2-COV (the virus that causes Covid-19)?<br>{[positive where] checkbox}<br>{Branching logic (show if): [positive] = '1' and [consent] = '1'}	☐ {5} Work (e.g., the school you teach at)<br>☐ {4} Home<br>☐ {3} Somewhere else<br>(Please select all that apply.)

If somewhere else, where do you think you were exposed?<br>{[positive somewhere] text}<br>{Branching logic (show if): [positive where(3)] = '1' and [consent] = '1'}	(Please describe.)
Do you understand the CURRENT Centers for Disease (CDC) guidelines for reducing Covid-19 transmission risk in my classroom?<br>{[cdc] radio}<br>{Branching logic (show if): [consent] = '1'}	◯ {1} Yes<br>◯ {0} No<br>◯ {-1} Do not know / Declined to answer<br>(Please select only one answer.)
Do you know who to ask at my school for any questions or concerns about Covid-19 guidance in my classroom?<br>{[questions] radio}<br>{Branching logic (show if): [consent] = '1'}	◯ {1} Yes<br>◯ {0} No<br>◯ {-1} Do not know / Declined to answer<br>(Please select only one answer.)
Are you immediately notified by your supervisor when a student you interacted with tests positive for COVID-19?<br>{[exposure] radio}<br>{Branching logic (show if): [consent] = '1'}	◯ {1} Yes<br>◯ {0} No<br>◯ {-1} Do not know / Declined to answer<br>(Please select only one answer.)
If yes, when you have interacted with a student who tested positive for Covid-19, did you have the ability to isolate at home for 10 days or quarantine when necessary without fear of repercussions from your supervisor?<br>{[isolate] radio}<br>{Branching logic (show if): [exposure] = '1' and [consent] = '1'}	◯ {1} Yes<br>◯ {0} No<br>◯ {-1} Do not know / Declined to answer<br>(Please select only one answer.)

Section C: Masks.

During the Covid-19 pandemic, personal mask wearing including N95, surgical or cloth masks were recommended by public health experts. Masks were also recommended by the Department of Education, Centers for Disease Control (CDC), the American Academy of Pediatrics and other government institutions to reduce transmission of Covid-19 spread. Mask mandates were subsequently adopted by school systems. The following section asks you questions about masks, mask-wearing and how this affected your classroom environment and teaching. Please answer each question to the best of your ability. All survey responses will be kept confidential and aggregated with other survey responses as to not personally identify you.

Thinking back to the last school year (2020-2021), what type of mask did you routinely wear to school during these times?<br>{[masktype] checkbox}<br>{Branching logic (show if): [consent] = '1'}	☐ {8} N95<br>☐ {7} KN95<br>☐ {6} Surgical mask<br>☐ {5} Cloth mask with insert filter<br>☐ {4} Cloth mask without insert filter<br>☐ {3} Double mask<br>☐ {2} Mask with clear window to see the mouth<br>☐ {1} Other<br>(Please select all that apply.)
If other, please specify:<br>{[masktype_other] text}<br>{Branching logic (show if): [masktype(1)] = '1' and [consent] = '1'}	(Please describe.)
If yes for N95, have you been fit tested (a 'fit test' tests the seal between the N95 mask's, or respirator's, facepiece and your face)?<br>{[n95_yes] radio}<br>{Branching logic (show if): [consent] = '1' and [masktype(8)] = '1'}	◯ {1} Yes<br>◯ {0} No<br>◯ {-1} Do not know / Declined to answer<br>(Please select only one answer.)
Thinking back to the last school year (2020-2021), did the mask or masks that you wore during school hours fit you well (i.e., covered the nose and chin with a good seal around your face)?<br>{[maskfit] radio}<br>{Branching logic (show if): [consent] = '1'}	◯ {1} Yes<br>◯ {0} No<br>◯ {-1} Do not know / Declined to answer<br>(Please select only one answer.)
Thinking back to the last school year (2020-2021), did you encounter problems/issues with compliance to the mask mandate with students in your classroom?<br>{[maskcomp] radio}<br>{Branching logic (show if): [consent] = '1'}	◯ {1} Yes<br>◯ {0} No<br>◯ {-1} Do not know / Declined to answer<br>(Please select only one answer.)
Thinking back to the last school year (2020-2021), what percentage or proportion of your students brought their own mask to wear in school (an estimate is fine).<br>{[maskpercent] text float}<br>{Branching logic (show if): [consent] = '1'}	(Please enter numbers only.)

Thinking back to the last school year (2020-2021), did your school district or school supply you (or your classroom) with extra masks?<br>{[masksextra] radio}<br>{Branching logic (show if): [consent] = '1'}	◯ {1} Yes<br>◯ {0} No<br>◯ {-1} Do not know / Declined to answer<br>(Please select only one answer.)
If yes, what size of masks did your school district/school supply you with?<br>{[masksize] radio}<br>{Branching logic (show if): [masksextra] = '1' and [consent] = '1'}	◯ {4} Adult-sized masks only<br>◯ {3} Child-sized masks only<br>◯ {2} Both adult and child-sized masks<br>◯ {1} Do not know / Declined to answer<br>(Please select only one answer.)
Thinking back to the last school year (2020-2021), at any time since the Covid-19 pandemic began, did you use personal funds to purchase extra masks for your classroom?<br>{[maskpurchase] radio}<br>{Branching logic (show if): [consent] = '1'}	◯ {1} Yes<br>◯ {0} No<br>◯ {-1} Do not know / Declined to answer<br>(Please select only one answer.)
Thinking back to the last school year (2020-2021), did you feel protected when you were NOT wearing a mask at work (i.e. school)?<br>{[masksafe] radio}<br>{Branching logic (show if): [consent] = '1'}	◯ {1} Yes<br>◯ {0} No<br>◯ {-1} Sometimes<br>◯ {-2} Do not know / Declined to answer<br>(Please select only one answer.)
Thinking back to the last school year (2020-2021), did you feel protected when your students did NOT wear masks to school?<br>{[maskstudentsafe] radio}<br>{Branching logic (show if): [consent] = '1'}	◯ {1} Yes<br>◯ {0} No<br>◯ {-1} Sometimes<br>◯ {-2} Do not know / Declined to answer<br>(Please select only one answer.)

Section D: Physical Distancing.

We are interested in some of the barriers to physical distancing guidelines experienced by schools during the COVID-19 Pandemic. CDC guidelines for physical distancing have changed over time (i.e., from 6ft. to 3 ft. distancing). The next section will ask you questions regarding physical distancing strategies used at your school and how these strategies have impacted your teaching of elementary aged children. Please answer each question to the best of your ability. All survey responses will be kept confidential and aggregated with other survey responses as to not personally identify you.

At the beginning of the 2021-2022 academic year (this school year), what was the total number of students taught in your classroom PER DAY?<br>{[no students day] radio}<br>{Branching logic (show if): [consent] = '1'}	◯ {3} < 15 students<br>◯ {2} 15-20 students<br>◯ {1} 21-40 students<br>◯ {0} >40 students<br>(Please select only one answer.)
At the beginning of the 2021-2022 academic year (this school year), what was the total number of students taught in your classroom PER WEEK?<br>{[no students week] radio}<br>{Branching logic (show if): [consent] = '1'}	◯ {3} < 15 students<br>◯ {2} 15-20 students<br>◯ {1} 21-40 students<br>◯ {0} >40 students<br>(Please select only one answer.)
During the 2021-2022 academic year (this school year), does your school use a "cohorting" or "classroom pod" strategy for physical distancing?<br>{[cohort] radio}<br>{Branching logic (show if): [consent] = '1' and [share] = '1'}	◯ {1} Yes<br>◯ {0} No<br>◯ {-1} Do not know / Declined to answer<br>(Please select only one answer.)
If yes, what are some of the "cohorting" or "classroom pod strategies" your school uses? (Please describe).<br><br>{[cohort yes] textarea}<br>{Branching logic (show if): [cohort] = '1'}	(Please describe.)
During the 2021-2022 academic year (this school year), does your classroom have enough space to maintain 3ft of physical distance between each of your students?<br>{[distance] radio}<br>{Branching logic (show if): [consent] = '1'}	◯ {1} Yes<br>◯ {0} No<br>◯ {2} Do not know / Declined to answer<br>(Please select only one answer.)
If no, what are some of the barriers that prohibits students from maintaining 3ft. physical distance?<br>{[distance no] textarea}<br>{Branching logic (show if): [distance] = '0' and [consent] = '1'}	(Please describe.)
During the 2021-2022 academic year (this school year), at any time during your typical school day do you share your classroom with another teacher?<br>{[share] radio}<br>{Branching logic (show if): [consent] = '1'}	◯ {1} Yes<br>◯ {0} No<br>◯ {3} Do not know / Declined to answer<br>(Please select only one answer.)

If yes, approximately how many hours per day do you share your classroom with another teacher?<br>{[share_yes] text}<br>{Branching logic (show if): [consent] = '1' and [share] = '1'}	((Hours))
During the 2021-2022 academic year (this school year), do you ever travel to other classrooms to teach?<br>{[travel] radio}<br>{Branching logic (show if): [consent] = '1'}	◯ {1} Yes<br>◯ {0} No<br>◯ {-1} Do not know / Declined to answer<br>(Please select only one answer.)
Do you have access to a teacher's lounge?<br>{[lounge_share] radio}<br>{Branching logic (show if): [consent] = '1'}	◯ {1} Yes<br>◯ {0} No<br>◯ {2} Do not know / Declined to answer<br>(Please select only one answer.)
If yes, do you share other items with teachers such as a microwave, table, chairs, printer or office supplies?<br>{[lounge_share_yes] radio}<br>{Branching logic (show if): [lounge_share] = '1' and [consent] = '1'}	◯ {1} Yes<br>◯ {0} No<br>◯ {-1} Do not know / Declined to answer<br>(Please select only one answer.)
If yes, are surfaces routinely cleaned/disinfected?<br>{[disinfected] radio}<br>{Branching logic (show if): [lounge_share_yes] = '1' and [consent] = '1'}	◯ {1} Yes<br>◯ {0} No<br>◯ {-1} Do not know / Declined to answer<br>(Please select only one answer.)

Section E: Surface Transmission Routes.

During the COVID-19 Pandemic, a major focus on reducing the risk of spread was placed on reducing surface transmission routes. The following section will ask you questions regarding the strategies used at your school to mitigate surface transmission routes. Please answer each question to the best of your ability. All survey responses will be kept confidential and aggregated with other survey responses as to not personally identify you.

During the 2021-2022 academic year (this school year), did you clean or disinfect the surfaces in your classroom?<br>{[disinfected classroom] radio}<br>{Branching logic (show if): [consent] = '1'}	◯ {1} Yes<br>◯ {0} No<br>◯ {2} Do not know / Declined to answer<br>(Please select only one answer.)
If yes, what types of products do you use? (Please describe).<br>{[products] textarea}<br>{Branching logic (show if): [disinfected classroom] = '1' and [consent] = '1'}	(Please describe.)
If yes, how often did you clean these surfaces?<br>{[disinfect classroom yes] radio}<br>{Branching logic (show if): [disinfected classroom] = '1' and [consent] = '1'}	◯ {3} 1-2 times a day<br>◯ {2} 3-4 times a day<br>◯ {1} >4 times a day<br>◯ {0} Do not know / Declined to answer<br>(Please select only one answer.)
When (at what times) did you clean these surfaces? (Please describe).<br>{[times clean] textarea}<br>{Branching logic (show if): [disinfected classroom] = '1' and [consent] = '1'}	(Please describe.)
Do you use hand sanitizer or have you used hand sanitizer stations in your classroom?<br>{[hand sanitizer] radio}<br>{Branching logic (show if): [consent] = '1' and [share] = '1'}	◯ {1} Yes<br>◯ {0} No<br>◯ {-1} Do not know / Declined to answer<br>(Please select only one answer.)
If yes, how often do you personally use hand sanitizer?<br>{[self sanitizer] radio}<br>{Branching logic (show if): [hand sanitizer] = '1' and [consent] = '1'}	◯ {3} 1-2 times a day<br>◯ {2} 3-4 times a day<br>◯ {1} >4 times a day<br>◯ {0} Do not know / Declined to answer<br>(Please select only one answer.)
If yes, when do you use hand sanitizer?<br>{[when sanitizer] checkbox}<br>{Branching logic (show if): [hand sanitizer] = '1' and [consent] = '1'}	☐ {1} After going to the bathroom<br>☐ {2} Before eating<br>☐ {3} After eating<br>☐ {4} Before I see my students<br>☐ {5} After I see my students<br>☐ {6} After touching a communal item<br>☐ {7} Other<br>(Please select all that apply.)
If other, please describe:<br>{[when sanitizer other] text}<br>{Branching logic (show if): [when sanitizer(7)] = '1' and [consent] = '1'}	(Please describe.)

Section F: Air Flow.

We are interested regarding the characteristics of your classroom environment including air flow and the ability to circulate air in your teaching environment. The following section will ask you questions regarding the strategies used at your school for increasing air flow. Please answer each question to the best of your ability. All survey responses will be kept confidential and aggregated with other survey responses as to not personally identify you.

During the 2021-2022 academic year (this school year), do you have windows that open in your classroom?<br>{[windows] radio}<br>{Branching logic (show if): [consent] = '1'}	◯ {1} Yes<br>◯ {0} No<br>◯ {-1} Sometimes<br>◯ {-2} Do not know / Declined to answer<br>(Please select only one answer.)
If yes, do you keep your windows open during class?<br>{[windows_yes] radio}<br>{Branching logic (show if): [windows] = '1' and [consent] = '1'}	◯ {1} Yes<br>◯ {0} No<br>◯ {-1} Sometimes<br>◯ {-2} Do not know / Declined to answer<br>(Please select only one answer.)
If no, what are some reasons you are unable to open the windows in your classroom?<br>{[windows_no] checkbox}<br>{Branching logic (show if): [consent] = '1' and [windows] = '0'}	☐ {1} There were no windows in my classroom<br>☐ {2} The windows in my classroom do not open<br>☐ {3} Climate control (Too hot or too cold if I opened the windows)<br>☐ {4} Outdoor air quality<br>☐ {5} Noise pollution<br>☐ {6} Opening the windows is distracting to my students<br>☐ {7} Safety concerns<br>☐ {8} There is a school policy that prohibits me from opening the windows<br>☐ {9} Other<br>(Please select all that apply.)
If other, please describe:<br>{[windows_other] textarea}<br>{Branching logic (show if): [windows_no(9)] = '1' and [consent] = '1'}	(Please describe.)
Do you keep your classroom door open during class or teaching time?<br>{[door_open] radio}<br>{Branching logic (show if): [consent] = '1'}	◯ {4} Yes<br>◯ {3} No<br>◯ {2} Sometimes<br>◯ {1} Do not know / Declined to answer<br>(Please select only one answer.)
Approximately what percent of the time?<br>{[door_open_sometimes] text}<br>{Branching logic (show if): [consent] = '1' and [door_open] = '4' and [door_open] = '2'}	(Please enter numbers only.)

If no, what are some of the reasons that you are unable to have your classroom door open?
{[door reasons] checkbox}
{Branching logic (show if): [door open] = '3' and [consent] = '1'}

- ☐ {8} My classroom door opens to the outside
- ☐ {7} Climate control (Too hot or too cold if I opened the door)
- ☐ {6} Outdoor air quality
- ☐ {5} Noise pollution
- ☐ {4} Opening the door is distracting to my students
- ☐ {3} Safety concerns
- ☐ {2} There is a school policy that prohibits me from opening the door
- ☐ {1} Other

(Please select all that apply.)

If other, please describe:
{[reasons other] textarea}
{Branching logic (show if): [door reasons(1)] = '1' and [consent] = '1'}

(Please describe.)

Section G: Contact Tracing Protocols.

The last section of questions will ask you about the contact tracing protocols for COVID-19 suspected cases used by your school. Please answer each question to the best of your ability. All survey responses will be kept confidential and aggregated with other survey responses as to not personally identify you.

During the 2021-2022 academic year (this school year), does your school/school district implement a contact tracing protocol for suspected COVID-19 individuals?<br>{[contact] radio}<br>{Branching logic (show if): [consent] = '1'}	◯ {1} Yes<br>◯ {0} No<br>◯ {-1} Do not know / Declined to answer<br>(Please select only one answer.)
During the 2021-2022 academic year (this school year), does your school have a designated health office for children who feel sick?<br>{[designated] radio}<br>{Branching logic (show if): [consent] = '1'}	◯ {1} Yes<br>◯ {0} No<br>◯ {-1} Do not know / Declined to answer<br>(Please select only one answer.)
During the 2021-2022 academic year (this school year), does your school have a separate place where children can go if they have COVID-19 symptoms?<br>{[separate] radio}<br>{Branching logic (show if): [consent] = '1'}	◯ {1} Yes<br>◯ {0} No<br>◯ {-1} Do not know / Declined to answer<br>(Please select only one answer.)
This is the second to the last survey question. Is there anything else that you would like to add regarding your experiences as a teacher during the Covid-19 pandemic?<br>{[comments] textarea}	(Please describe.)
[redacted]	◯ [redacted]<br>◯ [redacted]
